# Characterization of Imidazoline Receptors in Blood Vessels for the Development of Antihypertensive Agents

**DOI:** 10.1155/2014/182846

**Published:** 2014-04-03

**Authors:** Mei-Fen Chen, Jo-Ting Tsai, Li-Jen Chen, Tung-Pi Wu, Jia-Jang Yang, Li-Te Yin, Yu-lin Yang, Tai-An Chiang, Han-Lin Lu, Ming-Chang Wu

**Affiliations:** ^1^Department of Food Science, National Pingtung University of Science and Technology, Neipu, Pingtung 91201, Taiwan; ^2^College of Medicine and Life Science, Chung Hwa University of Medical Technology, Rende District, Tainan City 71703, Taiwan; ^3^Department of Radiation Oncology, Taipei Medical University-Shuang Ho Hospital, and College of Medicine, Taipei Medical University, Taipei City 10361, Taiwan; ^4^Institute of Basic Medical Sciences, College of Medicine, National Cheng Kung University, Tainan City 70101, Taiwan; ^5^Department of Obs/Gyn, Tainan Sin-Lau Hospital, The Presbyterian Church in Taiwan, Tainan City 70142, Taiwan; ^6^Department of Chinese Medicine, Tainan Sin-Lau Hospital, The Presbyterian Church in Taiwan, Tainan City 70142, Taiwan

## Abstract

It has been indicated that activation of peripheral imidazoline I_2_-receptor (I-2R) may reduce the blood pressure in spontaneously hypertensive rats (SHRs). Also, guanidinium derivatives show the ability to activate imidazoline receptors. Thus, it is of special interest to characterize the I-2R using guanidinium derivatives in blood vessels for development of antihypertensive agent(s). Six guanidinium derivatives including agmatine, amiloride, aminoguanidine, allantoin, canavanine, and metformin were applied in this study. Western blot analysis was used for detecting the expression of imidazoline receptor in tissues of Wistar rats. The isometric tension of aortic rings isolated from male rats was also estimated. The expression of imidazoline receptor on rat aorta was identified. However, guanidinium derivatives for detection of aortic relaxation were not observed except agmatine and amiloride which induced a marked relaxation in isolated aortic rings precontracted with phenylephrine or KCl. Both relaxations induced by agmatine and amiloride were attenuated by glibenclamide at concentration enough to block ATP-sensitive potassium (K_ATP_) channels. Meanwhile, only agmatine-induced relaxation was abolished by BU224, a selective antagonist of imidazoline I_2_-receptors. Taken together, we suggest that agmatine can induce vascular relaxation through activation of peripheral imidazoline I_2_-receptor to open K_ATP_ channels. Thus, agmatine-like compound has the potential to develop as a new therapeutic agent for hypertension in the future.

## 1. Introduction

Hypertension is known as the main risk parameters in patients with cardiovascular diseases, such as myocardial infarction and stroke. Many agents used in clinics are mentioned to produce side effects. Thus, development of the better agent to handle hypertension is urgent [1].

Imidazoline receptors are introduced to play a role in cardiovascular regulation [2, 3]. In recent, 3 subtypes of imidazoline receptors have been proposed; activation of I-1 receptors regulates the blood pressure through central nervous system [4], whereas I-3 receptors participate in insulin release [5] and activation of I-2 receptors (I-2R) increases glucose uptake into muscle cells [6, 7]. The clinical used antihypertensive agent rilmenidine may reduce blood pressure via an activation of imidazoline I_1_-receptors in brain to lower sympathetic tone [8, 9]. But, application of rilmenidine in hypertension is usually to produce some side effects such as mental depression, insomnia, and drowsiness. Thus, development of new agent for management of hypertension is essential. Recently, an activation of peripheral imidazoline I_2_-receptor (I-2R) was documented to produce antihypertensive actions in spontaneous hypertensive rats (SHRs) [10]. Thus, peripheral I-2R seems a potential target in development of antihypertensive drugs without side effects of sympathetic inhibition.

It has been documented that compounds with guanidine-like structures may bind to imidazoline receptors [11]. Thus, it is of special interest to investigate the effect of guanidinium derivatives on peripheral I-2R for vasodilatation. Then, this may help the development of new agent(s) for hypertension in the future.

## 2. Material and Methods

### 2.1. Animals

The male Wistar rats, weighing from 250 to 300 g, were obtained from the Animal Center of National Cheng Kung University Medical College. Animals were housed individually in plastic cages under standard laboratory conditions. We kept them under a 12 h light/dark cycle and had free access to food and water. All experiments were performed under anesthesia with 2% isoflurane to minimize the animals' suffering. The animal experiments were approved and conducted in accordance with local institutional guidelines for the care and use of laboratory animals, and the experiments conformed to the Guide for the Care and Use of Laboratory Animals as well as the guidelines of the Animal Welfare Act.

### 2.2. Preparation of Isolated Aortic Rings

Isolation of aortas was performed as described previously [10] from Wistar rats. After sacrifice under anesthesia with pentobarbital (50 mg/kg), the thoracic aortas were removed to put in the oxygenated Krebs' buffer (95% O_2_, 5% CO_2_). Aortas were cut into ring segments about 3 mm without fat and connective tissue. Then, as described previously [10], they were mounted in the organ baths containing 10 mL oxygenated Krebs' buffer (95% O_2_, 5% CO_2_) at 37°C.

Similar to previous report [10], each ring was connected to strain gauges (FT03; Grass Instrument, Quincy, MA, USA) to measure the isometric tension through chart software (MLS023, Powerlab; AD Instruments, Bella Vista, NSW, Australia). Samples were mounted to stabilize for 2 h. Each ring was then stretched gradually for optimal resting tension at 1 g.

### 2.3. Vasodilatation Caused by Guanidinium Derivatives

After the stabilization of resting tone, a solution of either phenylephrine (Sigma-Aldrich, St. Louis, MO, USA) or KCl prepared in distilled water was added to the bathing buffer to induce a marked raise in vascular tone followed by a stable vasoconstriction (tonic contraction). The final concentration in the organ bath of both phenylephrine and KCl was 1 *μ*mol/L and 50 mmol/L, similar to previous report [10]. Rings of the treated group were exposed to agmatine, amiloride, metformin, allantoin, canavanine, and aminoguanidine (10 *μ*M) for recording the alterations in tonic contraction (vasodilatation). Relaxation is expressed as the decreased percentage in maximal tonic contraction.

### 2.4. Effects of Blockers on Guanidinium Derivatives-Induced Vasodilatation

Aortic rings were exposed to BU224 (Research Biochemical, Wayland, MA, USA), a selective antagonist of imidazoline I_2_-receptors, for 15 min prior to the addition of guanidinium derivatives into the organ bath. Glibenclamide (Tocris Cookson, Bristol, UK), as blocker specific for K_ATP_ channels, was administered in the same manner. The changes of vasodilatation after treatment with inhibitor were compared to vehicle-treated groups.

### 2.5. Western Blotting Analysis

Western blotting analysis was performed as the previous method [10] and we extracted protein from tissue homogenates using ice-cold radioimmunoprecipitation assay (RIPA) buffer supplemented with phosphatase and protease inhibitors (50 mmol/L sodium vanadate, 0.5 mM phenylmethylsulphonyl fluoride, 2 mg/mL aprotinin, and 0.5 mg/mL leupeptin). Concentrations of protein were determined with a Bio-Rad protein assay (Bio-Rad Laboratories, Inc., Hercules, CA, USA). Total proteins (30 *μ*g) and were separated by SDS/polyacrylamide gel electrophoresis (10% acrylamide gel) using a Bio-Rad Mini-Protein II system. The protein was transferred to expanded polyvinylidene difluoride membranes (Pierce, Rockford, IL, USA) with a Bio-Rad Trans-Blot system. After transfer, the membranes were washed with PBS and blocked for 1 h at room temperature with 5% (w/v) skimmed milk powder in PBS. The manufacturer's instructions were followed for the primary antibody reactions. Following blocking, the blots were developed using antibodies for imidazoline receptors (IR) (Abcam, Cambridge, UK). The blots were subsequently hybridized using horseradish peroxidase-conjugated goat anti-goat IgG (Jackson ImmunoResearch Laboratories, Inc., PA, USA) and developed using the Western Lightning Chemiluminescence Reagent PLUS (PerkinElmer Life Sciences Inc., Boston, MA, USA). Densities of the obtained immunosblots at 37 KDa for imidazoline receptors (IR) and 43 KDa for actin were quantified using Gel-Pro analyser software 4.0 (Media Cybernetics, Silver Spring, MD, USA).

### 2.6. Statistical Analysis

Results were expressed as mean ± SE of each group. Statistical analysis was carried out using Student's *t*-test analysis. Statistical significance was set as *P* < 0.05.

## 3. Results

### 3.1. Identification of Imidazoline Receptor Expression in Tissues Using Western Blotting Analysis

**Figure 1:**
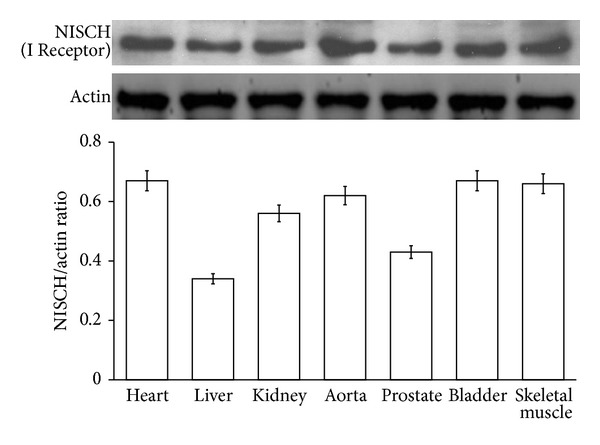
Detection of the expressions of imidazoline receptors in tissue homogenates by western blot analysis. The anti-NISCH (imidazoline receptors) antibody positively reacted with tissue lysate of heart, liver, aorta, skeletal muscle (SM), kidney, prostate, and bladder by western blot analysis. All values are presented as mean ± SEM (*n* = 8).

The anti-NISCH (imidazoline) antibody positively reacted with the tissue lysate prepared from heart, aorta, pancreas, skeletal muscle, kidney, prostate, and urinary bladder using western blotting analysis (Figure 1). The expression of imidazoline receptor in aorta can thus be identified.

### 3.2. Effects of Guanidinium Derivatives on Vascular Tone

**Figure 2:**
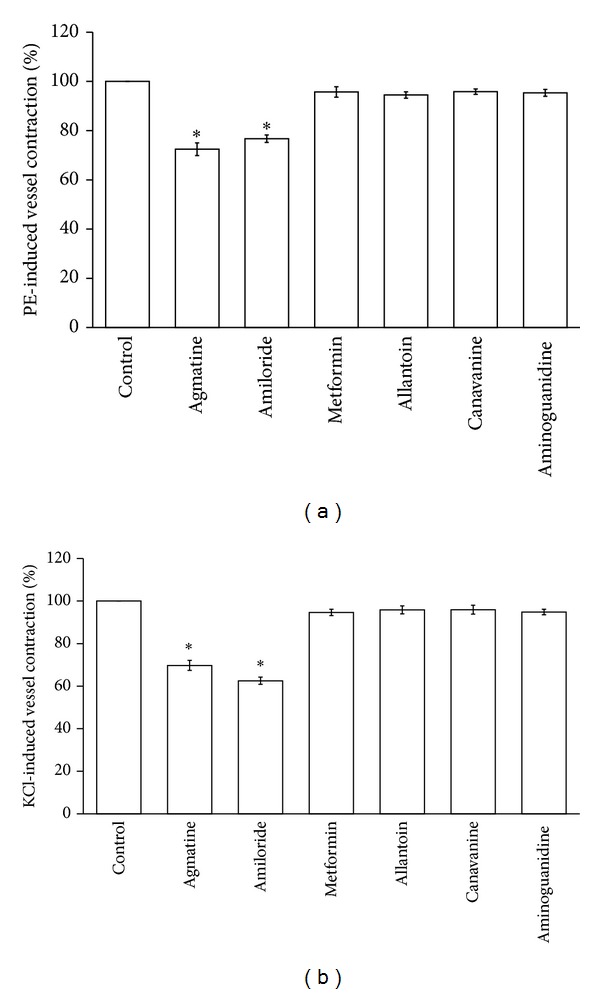
Screening of guanidinium derivatives for vasodilatation. Guanidinium derivatives (10 *μ*mol/L), agmatine, amiloride, metformin, allantoin, canavanine, and aminoguanidine induced relaxation in isolated aortic rings precontracted with 1 *μ*mol/L phenylephrine (a) or 50 mmol/L KCl (b). Data represent the mean ± SEM of eight animals in each column. All values are presented as mean ± SEM (*n* = 8). *P < 0.05 as compared to the precontracted value.

Six guanidinium derivatives of agmatine, amiloride, metformin, allantoin, canavanine, and aminoguanidine were tested in current study. Aortic ring strips are markedly contracted by the application of phenylephrine (1 *μ*mol/L) or KCl (50 mmol/L) as described previously [12]. Similar to the previous report [13], most guanidinium derivatives did not modify the vascular tone of aortic rings at the pharmacological concentration (10 *μ*mol/L) but agmatine and amiloride significantly relaxed the tonic contraction of rats' aortic rings induced by phenylephrine. Similarly, KCl-induced tonic vasoconstriction was relaxed by agmatine and amiloride (Figure 2).

### 3.3. Effects of Imidazoline Receptor Antagonism on Agmatine or Amiloride-Induced Vasodilatation

**Figure 3:**
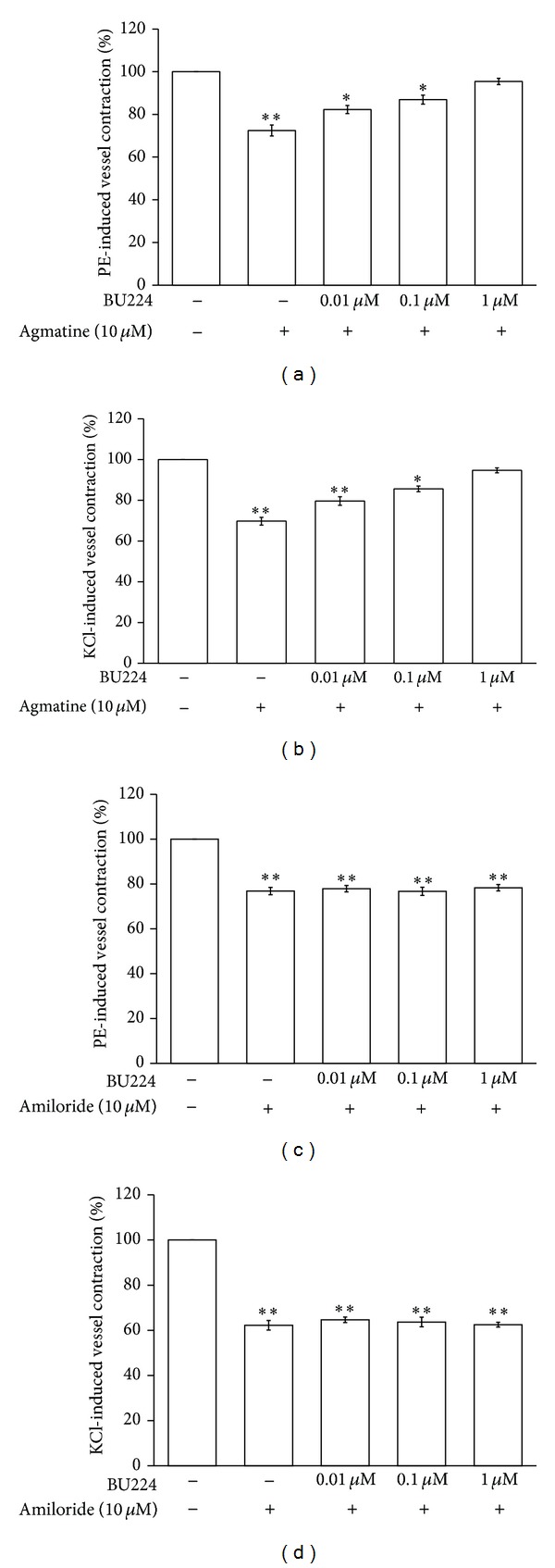
Effects of imidazoline receptor blockade on agmatine- or amiloride-induced vasodilatation. Effect of BU224 on concentration-dependent inhibition of agmatine- and amiloride- (10 *μ*mol/L) induced relaxation in isolated aortic rings precontracted with 1 *μ*mol/L phenylephrine ((a) and (b)) or 50 mmol/L KCl ((c) and (d)). All values are presented as mean ± SEM (*n* = 8). *P < 0.05 and **P < 0.01 as compared to agmatine-treated group.

Agmatine or amiloride at concentration of 10 *μ*mol/L attenuated the tonic contraction of aortic rings induced by phenylephrine similar to that observed in KCl-induced tonic vasoconstriction. BU224 (0.01–1 *μ*mol/L) produced a concentration-dependent inhibition of agmatine-induced relaxation in phenylephrine- or KCl-precontracted aortic rings (Figure 3(a)). However, BU224 did not modify the vascular relaxing action of amiloride (Figure 3(b)).

### 3.4. The Role of ATP-Sensitive K^+^ (K_ATP_) Channels in Agmatine- or Amiloride-Induced Vasodilatation

**Figure 4:**
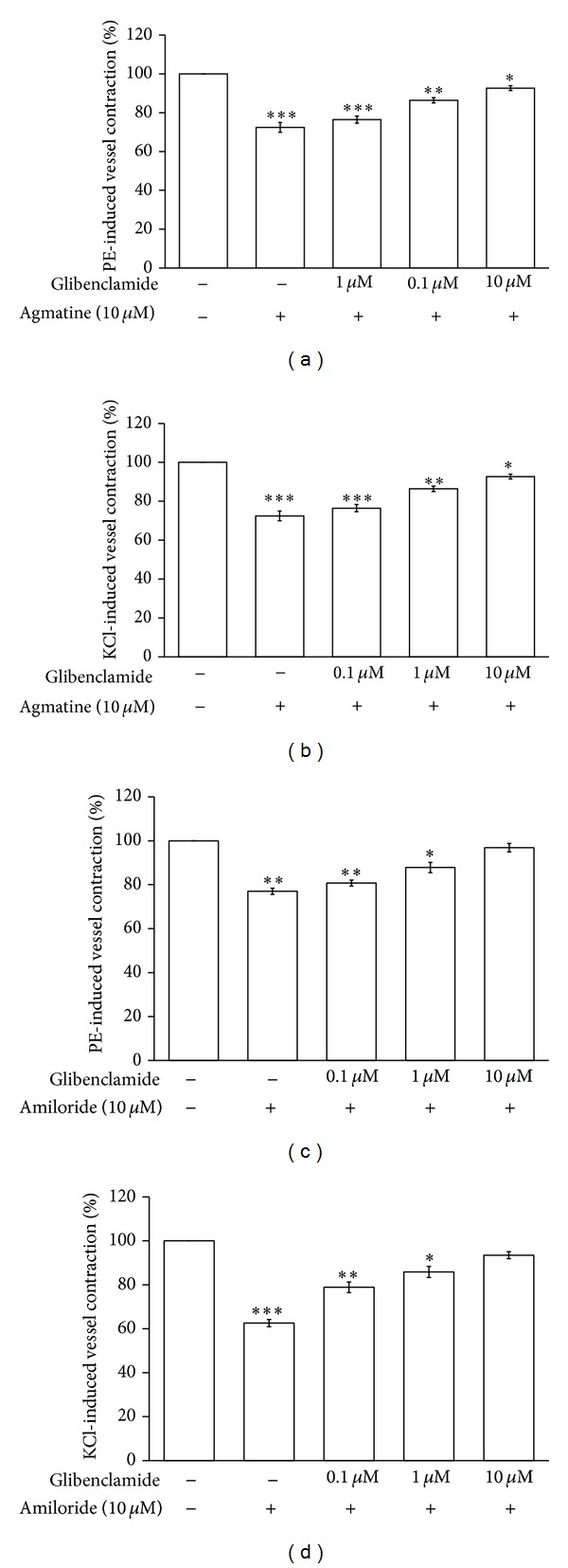
Effects of K_ATP_ blockade on agmatine- or amiloride-induced vasodilatation. Inhibitory effect of glibenclamide on the agmatine- or amiloride- (10 *μ*mol/L) induced relaxation in isolated aortic rings precontracted with 1 *μ*mol/L phenylephrine ((a) and (b)) or 50 mmol/L KCl (c and d). All values are presented as mean ± SEM (*n* = 8). *P < 0.05, **P < 0.01, and ***P < 0.001 as compared to the precontracted value.

Glibenclamide (0.1–10 nmol/L) produced a concentration-dependent inhibition of agmatine-induced vasodilatation in phenylephrine- or KCl-precontracted aortic rings (Figure 4(a)). Similar inhibitions were also observed in amiloride-induced vasodilatation (Figure 4(b)). However, as shown in these figures, glibenclamide at 10 nmol/L abolished amiloride-induced vasodilatation totally but not agmatine-induced vasodilatation.

### 3.5. Enhanced Vasodilatation in Aortic Rings by Cotreatment with Agmatine and Amiloride

**Figure 5:**
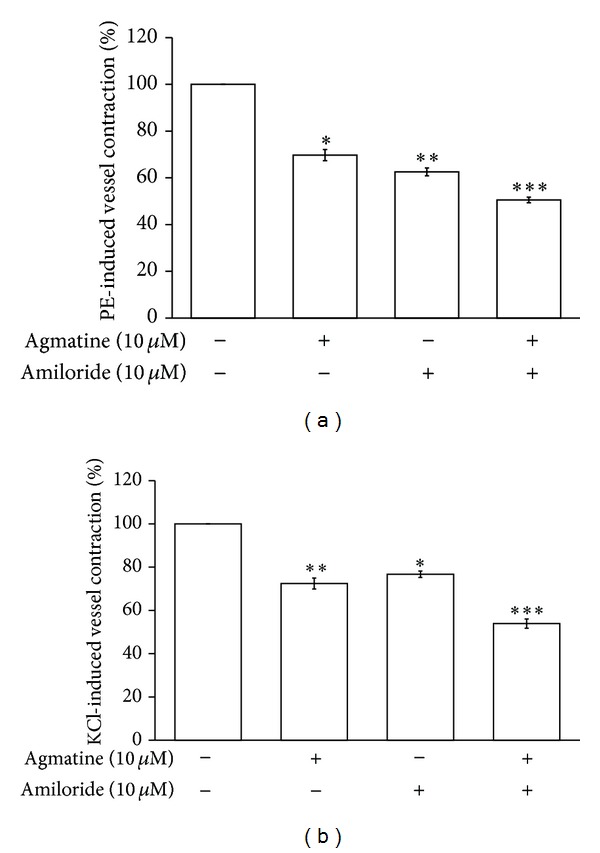
Vasodilatation in isolated aortic ring after cotreatment with agmatine and amiloride. Vasodilatation was enhanced by cotreatment with agmatine (10 *μ*mol/L) and amiloride (10 *μ*mol/L) in isolated aortic rings precontracted with 1 *μ*mol/L phenylephrine (a) or 50 mmol/L KCl (b). All values are shown as mean ± SEM (*n* = 8). **P < 0.01 and ***P < 0.001 as compared to the precontracted value.

At the maximal concentration (10 *μ*mol/L), agmatine or amiloride relaxed the tonic contraction of aortic rings induced by phenylephrine in a way similar to that in KCl-induced tonic vasoconstriction. The vasodilatation in aortic ring was enhanced by cotreatment with agmatine and amiloride (Figure 5).

## 4. Discussion

In the present study, we identified the expression of imidazoline receptor in aortic tissues isolated from rats. This is consistent with a previous report [10]. Then, we investigated the vasodilatation of guanidinium derivatives using agmatine, amiloride, metformin, allantoin, canavanine, and aminoguanidine. But only two agents, agmatine and amiloride, were effective for further characterizations. BU224, the well-known antagonist of imidazoline I_2_-receptors, showed no effect on amiloride-induced vascular relaxation at the dose effective to block agmatine-induced relaxation. Both agmatine- and amiloride-induced relaxations were attenuated by glibenclamide at concentration sufficient to block ATP-sensitive potassium (K_ATP_) channels. However, glibenclamide with the concentration effective to abolish amiloride-induced vasodilatation failed to block agmatine-induced vasodilatation totally (Figure 4). Also, enhanced vasodilatation was observed in aortic ring receiving the cotreatment with agmatine and amiloride at maximal concentration. Thus, agmatine-induced vascular relaxation seems not through the same mechanism as amiloride, while agmatine is known as the ligand of imidazoline receptors.

The imidazoline receptors are known to involve in cardiovascular regulations [14, 15]. Vascular tone is introduced as the main parameter in blood pressure regulations [16]. Actually, blood pressure is regulated by complicated factors; changes of blood pressure are widely used as the total peripheral resistance which is primarily a function of the resistance terminal arterioles [17]. Compounds with guanidine-like structures are known to bind with imidazoline receptors [11]. In this study, we employed six guanidinium derivatives including agmatine, amiloride, metformin, allantoin, canavanine, and aminoguanidine to screen the effects on vascular tone under the pharmacological dosage of 10 *μ*M. The results show that only agmatine and amiloride significantly relaxed the phenylephrine- or KCl-induced tonic contraction of aortic rings isolated from rats to consist with the previous reports [10, 18].

It has been mentioned that metformin can inhibit phenylephrine-mediated aortic contraction at the dosage of 2 mM [19]. It seems different with our results. Actually, the dosage used at 2 mM seems too high and the result is mostly considered as nonspecific action. Moreover, allantoin failed to produce vasodilatation in isolated aortic ring. This finding is further supporting our previous report showing that antihypertensive effect of allantoin is mainly through an activation of imidazoline receptor in central nervous system [20]. However, before now, no report mentioned the vasodilatation of canavanine or aminoguanidine. It is possible that the guanidine derivatives including canavanine and aminoguanidine may induce vasodilatation at the dose higher than that used in current study and this view needs more investigation in the future.

In an attempt to know the role of imidazoline I-2 receptor (I2-R) in agmatine- or amiloride-induced vasodilatations, imidazoline I-2R specific antagonist named BU224 was applied. Actually, the relaxation of agmatine was markedly reduced by pretreatment with BU224 at a concentration sufficient to block imidazoline I-2Rs. Thus, a direct effect of agmatine on I-2Rs can be identified. However, BU224 failed to modify the amiloride-induced vascular relaxation. The vessel dilatation of amiloride seems not to be through the activation of I-2Rs.

Due to a guanidino structure, amiloride binds to imidazoline I_2A_R through a high affinity for blockade of  I_2A_R at 0.1 *μ*M and inhibition of I_2B_R at a higher concentration of 2 *μ*M [21]. Thus, amiloride is widely used to distinguish the subtype of imidazoline I-2Rs. Previous study has also indicated that amiloride can induce vascular relaxation through an activation of Na^+^-H^+^ exchanger and consequently affect the K_ATP_ channel activity [22, 23]. In this study, BU224 failed to modify the 10 *μ*M amiloride-induced vascular relaxation. It is possible that vasodilatation induced by amiloride at the concentration of 10 *μ*M is mainly through the activation of K_ATP_ channels.

Potassium channels are mentioned as important in vascular relaxation [24]. ATP-sensitive potassium (K_ATP_) channels are known to have four inwardly rectifying K^+^ channel subunits and four regulatory sulfonylurea receptors [25]. Many contractions-induced endogenous substances are related to inhibition of K_ATP_ channels [25, 26]. Activation of K_ATP_ channels may produce hyperpolarization to relax vascular tone consequently. K_ATP_ channels dysfunction in aortic cells has been introduce to the impaired vasodilatation and/or hypertension observed in deoxycorticosterone acetate (DOCA) salt hypertensive rats [27]. In the present study, the relaxation induced by agmatine or amiloride in rat aortic rings was abolished by pretreatment with glibenclamide at a concentration sufficient to block K_ATP_ channels, as described previously [10, 23, 28, 29]. Thus, there is no doubt that K_ATP_ channels are involved in the aortic relaxation induced by agmatine or amiloride. However, agmatine-induced aortic relaxation seems not so simple although K_ATP_ channel is responsible for vasodilatation in PE- or KCl-induced contractions.

Agmatine has been introduced as an endogenous ligand of imidazoline receptors [30]. Activation of imidazoline I-2R by agmatine has also been mentioned in adrenal gland [31]. In this study, we demonstrated that BU224 at the dose enough to block imidazoline I-2Rs inhibited agmatine-induced vasodilatation. Thus, we suggest that agmatine has the ability to activate imidazoline I-2R on peripheral arterioles. As additional evidence, vasodilatation induced by agmatine at maximal dose was enhanced by cotreatment with amiloride. It means that amiloride has the ability to produce vasodilatation regardless of the total activation of imidazoline receptors. Amiloride seems not effective on imidazoline receptors only. Another mechanism for action of amiloride shall be investigated in the future.

## 5. Conclusion

According to the obtained data, we suggest that agmatine may act as peripheral antihypertensive agent through activation of imidazoline I-2 receptor for vasodilatation mainly through open of K_ATP_ channel. Thus, agmatine-like compound but not based on guanidinium structure has the potential to develop as a new antihypertensive agent in the future.
